# Comprehensive risk assessment for hospital-acquired pneumonia: sociodemographic, clinical, and hospital environmental factors associated with the incidence of hospital-acquired pneumonia

**DOI:** 10.1186/s12890-021-01816-9

**Published:** 2022-01-12

**Authors:** Bo-Guen Kim, Minwoong Kang, Jihyun Lim, Jin Lee, Danbee Kang, Minjung Kim, Jinhee Kim, Hyejeong Park, Kyung Hoon Min, Juhee Cho, Kyeongman Jeon

**Affiliations:** 1 Division of Pulmonary and Critical Care Medicine, Department of Medicine, Samsung Medical Center, Sungkyunkwan University School of Medicine, 81 Irwon-ro, Gangnam-gu, Seoul, 06351 South Korea; 2 Department of Digital Health, SAIHST, Sungkyunkawan University, Seoul, South Korea; 3 Center for Clinical Epidemiology, Samsung Medical Center, Seoul, South Korea; 4 Department of Clinical Research Design and Evaluation, SAIHST, Sungkyunkawan University, 115 Irwon-ro, Gangnam, Seoul, 06335 South Korea; 5 Division of Pulmonary, Allery, and Critical Care Medicine, Department of Internal Medicine, Korea University Guro Hospital, Seoul, South Korea

**Keywords:** Epidemiology, Hospital-acquired pneumonia, Risk factors, Mortality

## Abstract

**Background:**

Social and hospital environmental factors that may be associated with hospital-acquired pneumonia (HAP) have not been evaluated. Comprehensive risk assessment for the incidence of HAP including sociodemographic, clinical, and hospital environmental factors was conducted using national health insurance claims data.

**Methods:**

This is a population-based retrospective cohort study of adult patients who were hospitalized for more than 3 days from the Health Insurance Review and Assessment Service-National Inpatient Sample data between January 1, 2016 and December 31, 2018 in South Korea. Multivariable logistic regression analyses were conducted to identify the factors associated with the incidence of HAP.

**Results:**

Among the 512,278 hospitalizations, we identified 25,369 (5.0%) HAP cases. In multivariable analysis, well-known risk factors associated with HAP such as older age (over 70 vs. 20–29; adjusted odds ratio [aOR], 3.66; 95% confidence interval [CI] 3.36–3.99), male sex (aOR, 1.35; 95% CI 1.32–1.39), pre-existing lung diseases (asthma [aOR, 1.73; 95% CI 1.66–1.80]; chronic obstructive pulmonary disease [aOR, 1.62; 95% CI 1.53–1.71]; chronic lower airway disease [aOR, 1.79; 95% CI 1.73–1.85]), tube feeding (aOR, 3.32; 95% CI 3.16–3.50), suctioning (aOR, 2.34; 95% CI 2.23–2.47), positioning (aOR, 1.63; 95% CI 1.55–1.72), use of mechanical ventilation (aOR, 2.31; 95% CI 2.15–2.47), and intensive care unit admission (aOR, 1.29; 95% CI 1.22–1.36) were associated with the incidence of HAP. In addition, poverty (aOR, 1.08; 95% CI 1.04–1.13), general hospitals (aOR, 1.54; 95% CI 1.39–1.70), higher bed-to-nurse ratio (Grade ≥ 5; aOR, 1.45; 95% CI 1.32–1.59), higher number of beds per hospital room (6 beds; aOR, 3.08; 95% CI 2.77–3.42), and ward with caregiver (aOR, 1.19; 95% CI 1.12–1.26) were related to the incidence of HAP.

**Conclusions:**

The incidence of HAP was associated with various sociodemographic, clinical, and hospital environmental factors. Thus, taking a comprehensive approach to prevent and treat HAP is important.

**Supplementary Information:**

The online version contains supplementary material available at 10.1186/s12890-021-01816-9.

## Introduction

Hospital-acquired pneumonia (HAP) is one of the most common nosocomial infections [1, 2] and is associated with significant clinical and economic burdens, such as long-term hospitalization, high medical costs, and increased morbidity and mortality [3–7]. From studies conducted worldwide, its incidence ranges from five to more than 20 cases per 1000 hospital admissions and from 2.5 to more than 6.1 cases per 1000 patients not admitted to the intensive care unit (ICU) [5, 8, 9]. In addition, previous studies have found that older age and preexisting lung diseases, such as chronic obstructive pulmonary disease (COPD), asthma, and interstitial lung disease, or multiple organ system disorders increased the risk of HAP [6, 8]. Moreover, aspiration, intubation, and mechanical ventilation (MV) were risk factors for HAP [10, 11].

**Fig. 1:**
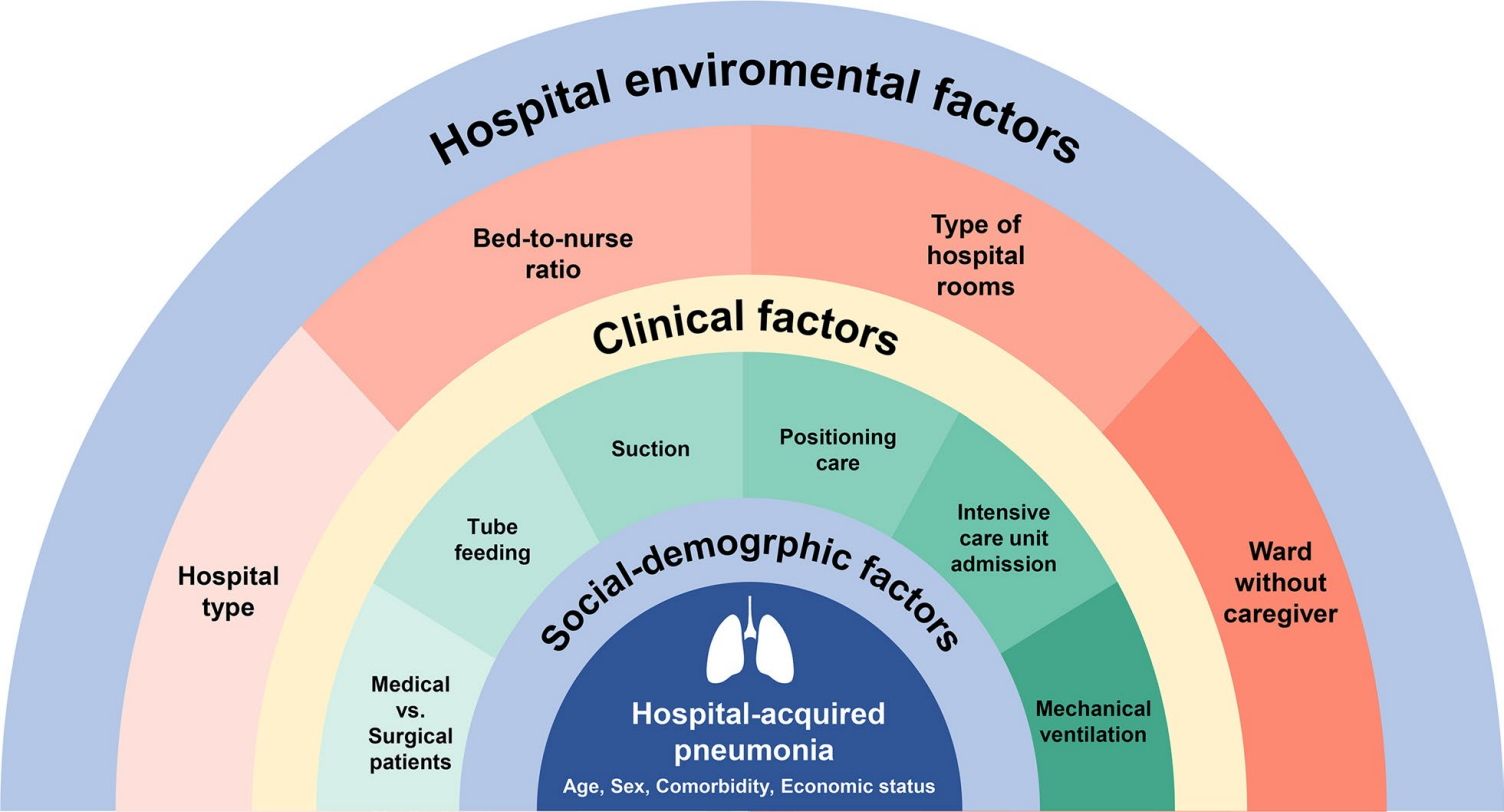
Comprehensive risk assessment for hospital-acquired pneumonia

Considering that HAP is an exogenous infection with nosocomial pathogens acquired from the hospital environment, evaluating hospital environment-related risk factors, such as hospital type, bed-to-nurse ratio, and hospital room type, would be necessary. However, studies on hospital environment-associated risk factors for HAP are limited. Furthermore, studies excluded poverty, which is a strong risk factor for other infectious diseases [12, 13]. Thus, we conducted a comprehensive risk assessment, including sociodemographic, clinical, and hospital environmental factors associated with the incidence of HAP (Fig. 1), using national health insurance claims data.

## Methods

This is a population-based retrospective cohort study based on data from the Health Insurance Review and Assessment Service-National Inpatient Sample (HIRA-NIS). We used the HIRA-NIS in 2016, 2017, and 2018, which included 13% of a representative sample of all inpatients in South Korea during the study period [14]. The inclusion criteria were men and women aged 18 years and older, who were hospitalized for more than 3 days in a tertiary or general hospital. If a patient had multiple inpatient records, we only considered the first episode. We excluded patients who had pneumonia within 3 months before hospitalization using codes in the 10th revision of the International Classification of Diseases (ICD). Additionally, we excluded patients who were admitted to the hospital from the emergency room and who were suspected of community-acquired pneumonia (CAP). The Institutional Review Board of Samsung Medical Center approved this study and waived the requirement for informed consent, as only de-identified data were used (SMC201912141-HE002).

### Measurement

We used claims data to define HAP. Patients who (1) underwent chest radiography, (2) were diagnosed with pneumonia on the same day, and (3) received antibiotics during hospitalization were considered patients with HAP. Additionally, we considered patients to have HAP if they were diagnosed with pneumonia within 3 days after discharge.

We included information on sociodemographic characteristics, comorbidities, procedures, prescriptions, and hospital characteristics based on claim codes. We used information on the type of health insurance to describe people living in poverty. Approximately 97.2% of the South Korean population was covered by the Korean National Health Insurance (KNHI), and the remaining 2.8% were covered by Medical Aid, which is a public assistance program targeted at poor individuals who are recipients of the National Basic Livelihood Security System based on the Medical Care Assistance Act [15]. For this study, we considered people with Medical Aid as people in poverty.

Comorbidities including asthma, COPD, other chronic lower respiratory diseases, chronic kidney disease (CKD), and anemia were defined as the presence of ICD-10 codes at admission and within 3 months before hospitalization. Procedures of interest during hospitalization included tube feeding, suctioning, positioning care, MV for more than 3 h, surgery, and ICU admissions. For hospital environment-related variables, the type and location of the hospital, the bed-to-nurse ratio, the type of hospital room, and ward with caregivers were considered. Hospitals were classified according to their capacity based on the number of hospital beds and specialties, as defined by the Korean Health Law [16]. General hospitals are defined as hospitals with more than 100 beds and at least seven specialty areas, and tertiary hospitals should have more than 500 beds with more than 20 specialty departments that serve as teaching hospitals to medical students and nurses. The location of the hospital was categorized as Seoul metropolitan, other metropolitan areas, and provinces. In 1999, the South Korean government implemented a new staffing policy that differentiates nursing fees for inpatients based on the bed-to-nurse ratio, from grade 1 to grade 7. The type of hospital room was based on the number of beds (patients) per room. Wards without caregivers are areas where patients are cared for by nursing staff alone and caregivers do not stay at the bedside. For the analysis of the type of hospital room and wards with caregivers, special units, such as the ICU, lead shield, and clean room, were excluded. Detailed codes for all variables in the additional Additional file 1: Table S1 (see Additional file 1).

### Statistical analyses

The means and standard deviations or medians and interquartile ranges were used to describe the distribution of continuous variables. To compare patients with and without HAP, a t-test for continuous variables and the chi-square test for categorical variables were used. We performed univariate and multivariate logistic regression analyses to identify the factors associated with HAP. We used the hospital as a random intercept in the mixed-effects logistic model. Odds ratios (ORs) with 95% confidence intervals (CIs) were estimated using the models. We performed mixed-effects logistic regression using the PROC GLIMMIX procedure in SAS (SAS Institute, Inc., North Carolina, USA). For the multivariable model, we adjusted for age, sex, poverty, asthma, COPD, other chronic lower respiratory diseases, CKD, anemia, tube feeding, suctioning, positioning, surgery, MV, year of hospitalization, hospital location, and hospital type. Additionally, we performed a subgroup analysis for medical and surgical patients. All analyses were performed using SAS (version 9.4; SAS Institute, Inc., North Carolina, USA). *P* values of less than 0.05 were used to denote statistical significance.

## Results

### Baseline characteristics

**Fig. 2:**
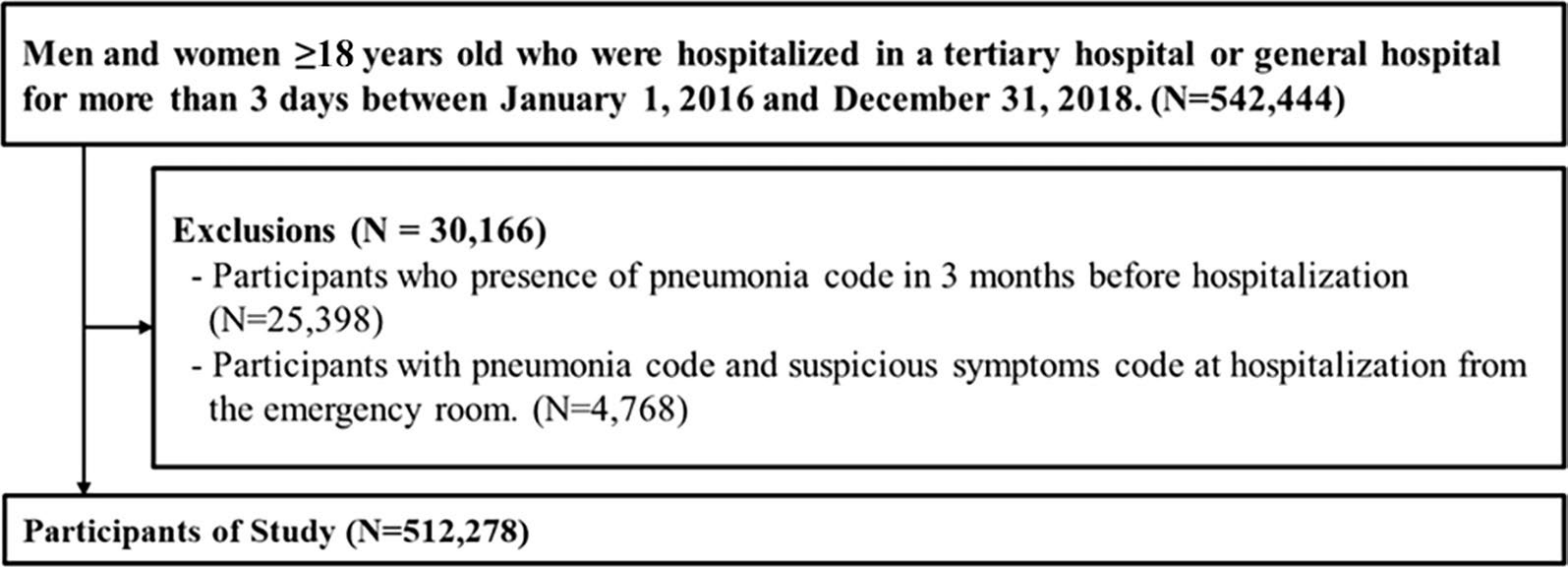
Flow chart of the study participants

Between January, 2016 and December, 2018, 542,444 patients were identified. Patients with pneumonia codes 3 months before hospitalization (n = 25,398) and patients with pneumonia and suspicious symptom codes at hospitalization from the emergency room (n = 4,768) were excluded; the remaining 512,278 patients were included in the final sample (Fig. 2).

Among the 512,278 patients, 25,369 (5.0%) had HAP. The characteristics of the patients with HAP are presented in Table 1. The elderly group aged 70 years and older had a higher rate of HAP (57.0%) than the no HAP group (28.0%) (*p* < 0.001). Regarding comorbidity, patients with HAP had a higher proportion of each comorbidity than those without HAP, and other chronic lower respiratory diseases (23.9%) were the highest in the HAP group. In the procedures of interest during hospitalization, tube feeding (18.3% vs. 2.2%), suctioning (20.0% vs. 4.1%), positioning care (25.8% vs. 7.1%), MV (11.0% vs. 1.3%), and ICU admission (27.3% vs. 9.0%) were more frequent in the HAP group than in the no HAP group.

**Table 1:** Characteristics of the study participants (N = 512,278)

Variables	No HAP (n = 486,909)	HAP (n = 25,369)	*p* value
Age group			< 0.001
20–29	35,400 (7.3)	604 (2.4)	
30–39	50,320 (10.3)	1022 (4.0)	
40–49	68,073 (14.0)	1610 (6.4)	
50–59	100,721 (20.7)	3191 (12.6)	
60–69	96,000 (19.7)	4495 (17.7)	
Over 70	136,395 (28.0)	14,447 (57.0)	
Sex (male)	224,522 (46.1)	13,655 (53.8)	< 0.001
Poverty (yes)	38,330 (7.9)	3122 (12.3)	< 0.001
Asthma (yes)	33,331 (6.9)	4485 (17.7)	< 0.001
COPD (yes)	14,412 (3.0)	2491 (9.8)	< 0.001
Other chronic lower respiratory disease (yes)	52,745 (10.8)	6053 (23.9)	< 0.001
CKD (yes)	18,098 (3.7)	1473 (5.8)	< 0.001
Anemia (yes)	39,829 (8.2)	2723 (10.7)	< 0.001
Tube feeding (yes)	10,663 (2.2)	4634 (18.3)	< 0.001
Suctioning (yes)	19,773 (4.1)	5061 (20.0)	< 0.001
Positioning (yes)	34,588 (7.1)	6534 (25.8)	< 0.001
Surgery (yes)	189,888 (39.0)	4430 (17.5)	< 0.001
Mechanical ventilation (yes)	6295 (1.3)	2785 (11.0)	< 0.001
ICU admission (yes)	43,645 (9.0)	6935 (27.3)	< 0.001
Location of hospital			< 0.001
Seoul metropolitan area	204,419 (42.0)	8997 (35.5)	
Other metropolitan area	142,073 (29.2)	7218 (28.5)	
Province	140,417 (28.8)	9154 (36.0)	
Type of hospital			< 0.001
Tertiary	172,295 (35.4)	6344 (25.0)	
General	314,614 (64.6)	19,025 (75.0)	
Bed-to-nurse ratio^*^(n = 425,953)			< 0.001
Grade 1	79,427 (19.6)	2671 (12.6)	
Grade 2	157,134 (38.3)	7564 (35.5)	
Grade 3	72,569 (17.9)	4291 (20.2)	
Grade 4	21,210 (5.2)	1441 (6.8)	
Grade $\ge$ 5	74,329 (18.4)	5317 (25.0)	
Types of hospital rooms (n = 504,279)			< 0.001
≤ 3 beds	44,027 (9.2)	383 (1.6)	
4 beds	68,876 (14.3)	3816 (16.2)	
5 beds	134,363 (28.0)	7096 (30.1)	
6 beds	233,445 (48.6)	12,273 (52.1)	
Ward with or without caregiver^†^ (n = 469,588)			0.498
With caregivers	404,669 (90.7)	21,284 (90.8)	
Without carefgivers	41,487 (9.3)	2148 (9.2)	
Year			< 0.001
2016	185,262 (38.1)	10,372 (40.9)	
2017	148,876 (30.6)	7624 (30.1)	
2018	152,771 (91.4)	7373 (29.1)	

The values in the table are numbers (percentages)

*CKD* chronic kidney disease, *COPD* chronic obstructive pulmonary disease, *HAP* hospital-acquired pneumonia, *ICU* intensive care unit

^*^Bed-to-nurse ratio grading is defined as follows: tertiary hospitals are divided into the following grades: grade 1 (a bed-to-nurse ratio of less than 2.0), grade 2 (a bed-to-nurse ratio of 2.0–2.4), grade 3 (a bed-to-nurse ratio of 2.5–2.9), grade 4 (a bed-to-nurse ratio of 3.0–3.4), grade 5 (a bed-to-nurse ratio of 3.5–3.9), and grade 6 (a bed-to-nurse ratio of 4.0 or more). General hospitals are classified into the following grades: grade 1 (a bed-to-nurse ratio of less than 2.5), grade 2 (a bed-to-nurse ratio of 2.5–2.9), grade 3 (a bed-to-nurse ratio of 3.0–3.4), grade 4 (a bed-to-nurse ratio of 3.5–3.9), grade 5 (a bed-to-nurse ratio of 4.0–4.4), grade 6 (a bed-to-nurse ratio of 4.5–5.9), and grade 7 (a bed-to-nurse ratio of 6.0 or more)

^**†**^Ward without caregiver is where patients are cared for by the nursing staff only, and caregivers do not stay at the bedside

### Risk factors associated with the incidence of HAP

**Fig. 3:**
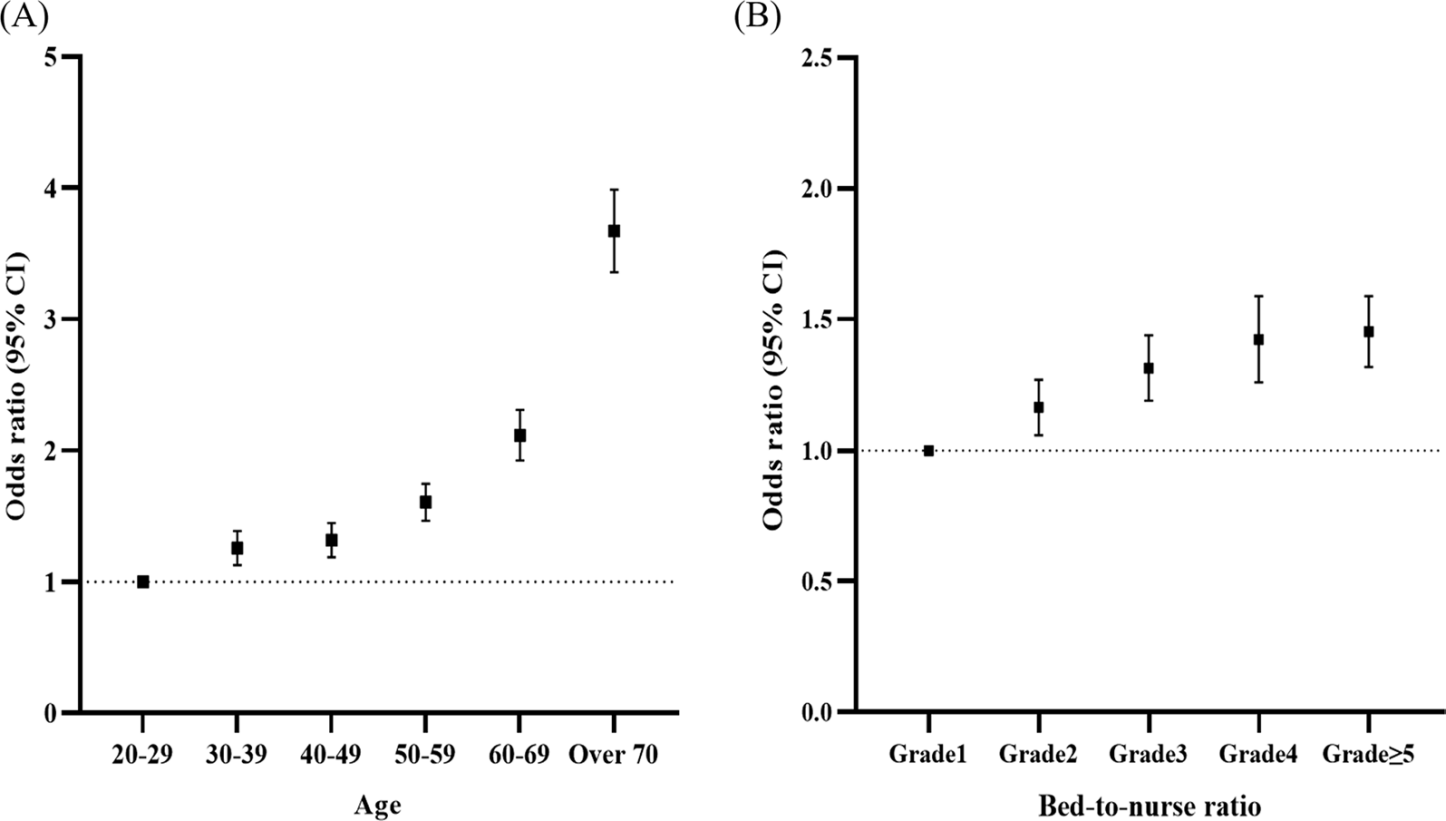
The odds ratio of hospital-acquired pneumonia incidence according to **A** age and **B** bed-to-nurse ratio

In multivariable analysis, old age (over 70 vs. 20–29; adjusted OR, 3.66; 95% CI 3.36–3.99), male sex (adjusted OR, 1.35; 95% CI 1.32–1.39), poverty (adjusted OR, 1.08; 95% CI 1.04–1.13), asthma (adjusted OR, 1.73; 95% CI 1.66–1.80), COPD (adjusted OR, 1.62; 95% CI 1.53–1.71), other chronic lower respiratory diseases (adjusted OR, 1.79; 95% CI 1.73–1.85), and CKD (adjusted OR, 1.07; 95% CI 1.00–1.14) were risk factors associated with the incidence of HAP (Table 2). The OR of HAP occurrence tended to increase as the age group increased (Fig. 3A). The association of various comorbidities with HAP occurrence are detailed in Additional file 2: Table S2 (see Additional file 2). Dementia (adjusted OR, 1.32; 95% CI 1.27–1.38), paraplegia and hemiplegia (adjusted OR, 1.15; 95% CI 1.05–1.25), and metastatic carcinoma (adjusted OR, 1.15; 95% CI 1.06–1.25) were associated with the occurrence of HAP.

**Table 2:** Factors associated with the incidence of hospital-acquired pneumonia

Characteristics	Univariable	Multivariable
	OR (95% CI)	Adjusted OR (95% CI)
Age group		
20–29	*Reference*	*Reference*
30–39	1.25 (1.13–1.38)	1.25 (1.13–1.39)
40–49	1.42 (1.29–1.56)	1.31 (1.19–1.45)
50–59	1.91 (1.75–2.09)	1.60 (1.47–1.75)
60–69	2.91 (2.67–3.17)	2.11 (1.93–2.31)
Over 70	6.22 (5.73–6.76)	3.66 (3.36–3.99)
Sex (male)	1.36 (1.32–1.39)	1.35 (1.32–1.39)
Poverty (yes)	1.45 (1.39–1.51)	1.08 (1.04–1.13)
Asthma (yes)	2.83 (2.73–2.93)	1.73 (1.66–1.80)
COPD (yes)	3.60 (3.44–3.77)	1.62 (1.53–1.71)
Other chronic lower respiratory disease (yes)	2.56 (2.48–2.64)	1.79 (1.73–1.85)
CKD (yes)	1.71 (1.62–1.81)	1.07 (1.00–1.14)^*^
Anemia (yes)	1.42 (1.36–1.48)	1.04 (1.00–1.10)^†^
Tube feeding (yes)	11.25 (10.82–11.71)	3.32 (3.16–3.50)
Suction (yes)	7.15 (6.89–7.42)	2.34 (2.23–2.47)
Positioning (yes)	4.71 (4.57–4.86)	1.63 (1.55–1.72)
Surgery (no)	2.76 (2.67–2.85)	2.98 (2.87–3.09)
Mechanical ventilation (yes)	11.40 (10.85–11.98)	2.31 (2.15–2.47)
ICU admission (yes)	4.22 (4.09–4.35)	1.29 (1.22–1.36)
Location of hospital		
Seoul metropolitan area	*Reference*	*Reference*
Other metropolitan area	1.09 (1.05–1.14)	1.16 (1.06–1.26)
Province	1.40 (1.35–1.45)	1.20 (1.11–1.31)
Type of hospital		
Tertiary	*Reference*	*Reference*
General	1.53 (1.37–1.69)	1.54 (1.39–1.70)
Bed-to-nurse ratio^‡^ (n = 425,953)		
Grade 1	*Reference*	*Reference*
Grade 2	1.12 (1.03–1.23)	1.16 (1.06–1.27)
Grade 3	1.36 (1.24–1.50)	1.31 (1.19–1.44)
Grade 4	1.59 (1.42–1.78)	1.42 (1.26–1.59)
Grade $\ge$ 5	1.62 (1.49–1.77)	1.45 (1.32–1.59)
Type of hospital room (n = 504,279)		
≤ 3 beds	*Reference*	*Reference*
4 beds	5.38 (4.83–5.99)	3.26 (2.92–3.64)
5 beds	6.08 (5.48–6.76)	3.34 (3.00–3.72)
6 beds	5.10 (4.60–5.65)	3.08 (2.77–3.42)
Ward with or without caregiver^§^ (n = 469,588)		
With caregivers	1.09 (1.03–1.14)	1.19 (1.12–1.26)
Without caregivers	*Reference*	*Reference*

The multivariable analysis included age, sex, poverty, asthma, COPD, other chronic lower respiratory diseases, CKD, anemia, tube feeding, suctioning, positioning, surgery, mechanical ventilation, ICU admission, year of hospitalization, location of the hospital, and type of hospital

CI confidence interval; CKD, chronic kidney disease; COPD, chronic obstructive pulmonary disease; HAP, hospital-acquired pneumonia; ICU, intensive care unit; OR, odds ratio

^*^p = 0.03

^**†**^*p* = 0.08

^**‡**^Bed-to-nurse ratio grading was defined as follows: tertiary hospitals were divided into the following grades: grade 1 (a bed-to-nurse ratio of less than 2.0), grade 2 (a bed-to-nurse ratio of 2.0–2.4), grade 3 (a bed-to-nurse ratio of 2.5–2.9), grade 4 (a bed-to-nurse ratio of 3.0–3.4), grade 5 (a bed-to-nurse ratio of 3.5–3.9), and grade 6 (a bed-to-nurse ratio of 4.0 or more). General hospitals are classified into the following grades: grade 1 (a bed-to-nurse ratio of less than 2.5), grade 2 (a bed-to-nurse ratio of 2.5–2.9), grade 3 (a bed-to-nurse ratio of 3.0–3.4), grade 4 (a bed-to-nurse ratio of 3.5–3.9), grade 5 (a bed-to-nurse ratio of 4.0–4.4), grade 6 (a bed-to-nurse ratio of 4.5–5.9), and grade 7 (a bed-to-nurse ratio of 6.0 or more)

^**§**^In wards without caregivers, only the nursing staff takes care of the patients, and caregivers do not stay at the bedside

Among procedures during hospitalization, tube feeding (adjusted OR, 3.32; 95% CI 3.16–3.50), suction (adjusted OR, 2.34; 95% CI 2.23–2.47), and positioning care (adjusted OR, 1.63; 95% CI 1.55–1.72) were risk factors for HAP (Table 2). Medical patients (adjusted OR, 2.98; 95% CI 2.87–3.09) had a higher risk of HAP than surgical patients. Additionally, MV (adjusted OR, 2.31; 95% CI 2.15–2.47) and ICU admission (adjusted OR, 1.29; 95% CI 1.22–1.36) increased the risk of HAP (Table 2). As the bed-to-nurse ratio grade increased, the incidence of HAP increased (Fig. 3B). Six patients sharing one hospitalization room increased the risk of developing HAP (adjusted OR, 3.08; 95% CI 2.77–3.42) compared with using one hospitalization room with three or fewer patients. Patients hospitalized in a ward with caregivers (adjusted OR, 1.19; 95% CI 1.12–1.26) were at a higher risk of developing HAP than those admitted in a ward without caregivers.

We conducted a subgroup analysis involving medical and surgical patients (Table 3). Among surgical patients, those aged over 70 years were at a 6.7 times higher risk of HAP than those aged 20–29 years. However, ward with caregivers was not a significant factor for HAP in surgical patients.

**Table 3:** Odds ratios (95% confidence intervals) for risk factor with hospital-acquired pneumonia during hospitalization in medical and surgical patients

Characteristics	Medical	Surgical
	Adjusted OR (95% CI)	Adjusted OR (95% CI)
Age group		
20–29	*Reference*	*Reference*
30–39	1.26 (1.13–1.40)	1.32 (0.97–1.80)
40–49	1.26 (1.14–1.40)	1.90 (1.44–2.52)
50–59	1.53 (1.39–1.68)	2.37 (1.81–3.09)
60–69	2.01 (1.83–2.20)	3.19 (2.45–4.15)
Over 70	3.34 (3.06–3.66)	6.70 (5.17–8.70)
Sex, male	1.33 (1.29–1.37)	1.47 (1.37–1.57)
Poverty, yes	1.03 (0.99–1.08)	1.45 (1.31–1.60)
Asthma, yes	1.77 (1.69–1.85)	1.41 (1.27–1.58)
COPD, yes	1.70 (1.60–1.80)	1.20 (1.05–1.37)
Other chronic lower respiratory disease, yes	1.86 (1.79–1.93)	1.34 (1.22–1.47)
CKD, yes	1.00 (0.93–1.07)	1.34 (1.16–1.54)
Anemia, yes	1.01 (0.96–1.07)	1.20 (1.08–1.35)
Tube feeding, Yes	3.01 (2.84–3.20)	4.21 (3.82–4.64)
Suction, Yes	2.40 (2.25–2.55)	2.10 (1.92–2.31)
Positioning care, Yes	1.71 (1.61–1.81)	1.48 (1.34–1.63)
Mechanical ventilation, Yes	1.76 (1.61–1.93)	2.06 (1.85–2.29)
ICU admission	1.05 (0.99–1.12)	2.40 (2.17–2.67)
Location of hospital		
Seoul metropolitan area	*Reference*	*Reference*
Other metropolitan area	1.16 (1.06–1.27)	1.11 (1.02–1.21)
Province	1.17 (1.08–1.27)	1.42 (1.31–1.54)
Type of hospital		
Tertiary	*Reference*	*Reference*
General	1.60 (1.45–1.77)	1.27 (1.18–1.37)
Bed-to-nurse ratio^*^ (n = 425,953)		
Grade1	*Reference*	*Reference*
Grade2	1.22 (1.11–1.34)	1.09 (0.92–1.29)
Grade3	1.34 (1.21–1.48)	1.27 (1.05–1.54)
Grade4	1.42 (1.26–1.60)	1.46 (1.13–1.88)
Grade $\ge$ 5	1.43 (1.29–1.58)	1.71 (1.44–2.04)
Hospitalization room (n = 504,279)		
≤ 3 beds	*Reference*	*Reference*
4 beds	3.22 (2.86–3.62)	3.65 (2.63–5.09)
5 beds	3.22 (2.87–3.61)	3.91 (2.82–5.41)
6 beds	3.06 (2.74–3.42)	3.53 (2.55–4.87)
Ward with or without caregiver^†^ (n = 469,588)		
With caregivers	1.17 (1.10–1.25)	1.15 (0.98–1.34)
Without caregivers	*Reference*	*Reference*
Year		
2016	1.30 (1.19–1.42)	1.27 (1.17–1.37)
2017	1.17 (1.01–1.27)	1.09 (1.00–1.19)
2018	*Reference*	*Reference*

Multivariable analysis was including age, sex, poverty, asthma, COPD, other chronic lower respiratory diseases, CKD, anemia, tube feeding, suctioning, positioning, surgery, mechanical ventilation, ICU admission, year of hospitalization, location of hospital, and type of hospital

CI confidence interval; CKD, chronic kidney disease; COPD, chronic obstructive pulmonary disease; HAP, hospital-acquired pneumonia; ICU, intensive care unit; OR, odds ratio

^*^Bed-to-nurse ratio grade is defined as follows: tertiary hospitals are divided into the following grades: grade 1 (a bed-to-nurse ratio of less than 2.0), grade 2 (a bed-to-nurse ratio of 2.0–2.4), grade 3 (a bed-to-nurse ratio of 2.5–2.9), grade 4 (a bed-to-nurse ratio of 3.0–3.4), grade 5 (a bed-to-nurse ratio of 3.5–3.9), and grade 6 (a bed-to-nurse ratio of 4.0 or more). General hospitals are classified into the following grades: grade 1 (a bed-to-nurse ratio of less than 2.5), grade 2 (a bed-to-nurse ratio of 2.5–2.9), grade 3 (a bed-to-nurse ratio of 3.0–3.4), grade 4 (a bed-to-nurse ratio of 3.5–3.9), grade 5 (a bed-to-nurse ratio of 4.0–4.4), grade 6 (a bed-to-nurse ratio of 4.5–5.9), and grade 7 (a bed-to-nurse ratio of 6.0 or more)

^**†**^Ward without caregiver is where patients are cared for by the nursing staff only, and caregivers do not stay at the bedside

## Discussion

In this study, the incidence of HAP over 3 years was 5.0%, and older age, male sex, asthma, COPD, other chronic lower respiratory diseases, CKD, and poverty were associated with the incidence of HAP. Additionally, clinical factors, such as tube feeding, suctioning, positioning, MV, and ICU admission, increased the risk of HAP. In terms of the hospital environment, hospital type, beds-to-nurse ratio, hospital room type, and ward with caregivers were associated with the incidence of HAP.

Similar to previous studies, respiratory-related comorbidity, CKD, and age were risk factors associated with the incidence of HAP in this study [8, 17, 18]. According to a study conducted at a 1,000-bed hospital, patients aged over 60 years had a 2.8-fold higher risk of HAP than those aged below 60 years [19]. While approximately half of patients with HAP are aged below 60 years [20], evidence on how age is associated with increased risk of HAP in patients aged below 60 years is limited. In this study, we found a linear association between age and the incidence of HAP. Compared with patients aged 20–29 years, those in their 30 s, 40 s, and 50 s had 1.25-, 1.31-, and 1.60-fold higher risks, respectively.

Poverty and infectious diseases interact in complex ways [21], and poverty is a well-known risk factor for community-acquired pneumonia [12, 13]. According to previous studies, poor individuals have a higher risk of community-acquired pneumonia as they are more likely to have uncontrolled chronic diseases and less likely to have sufficient medical resources and access to care, resulting in longer hospital stays and higher mortality [13, 22, 23]. In this study, patients in poverty had a slightly higher risk of HAP than those not in poverty. In South Korea, through the KNHI, all registered citizens have access to care, and few health inequalities exist in South Korea [24]. Therefore, poverty might have a greater impact on the incidence of HAP in patients in other countries where there are larger differences in access to healthcare.

Studies have suggested that HAP is more commonly observed in medical patients than in surgical patients [8, 20], and we had similar findings. This might be because patients who are hospitalized for surgery would have sufficient health status to receive surgery than those who are hospitalized for medical problems [25]. However, this does not mean that surgical patients do not have the risk of HAP. Approximately one-fifth of patients with HAP in this study were surgical patients and had different risk factors for HAP. Older age had a greater impact on the incidence of HAP in surgical patients than in medical patients. Compared with patients aged 20–29 years (among surgical patients), those aged over 70 years were at a 6.7-fold higher risk of HAP, which was much higher than that in medical patients. Clinicians should pay more attention to older patients undergoing surgery to prevent and manage HAP.

In this study, the incidence of HAP in tertiary hospitals was 3.5%, whereas that in general hospitals was 5.7%. Similarly, the incidence of HAP in hospitals in Seoul was 4.2%; however, the incidence of HAP in the province was 6.1%. This difference could be due to differences in health resource access and quality of patient care [26]. Tertiary hospitals would have a better hygiene environment and better trained healthcare professionals associated with better quality care than those of general hospitals [27, 28]. Additionally, the bed-to-nurse ratio, which is one of the quality indicators for nursing care, was associated with the incidence of HAP. Patients who stayed in hospitals with grade 4 and 5 bed-to-nurse ratios had a 1.4-fold higher risk of HAP than those in the hospital with a grade 1 bed-to-nurse ratio. According to the literature, nursing quality and time have a direct impact on patient outcomes and the incidence of hospital-acquired infection [29–31]. A study found that a higher proportion of total hours of nursing care provided by registered nurses was 0.59 times lower than the incidence of HAP in medical patients [30]. Nurses would be able to spend more time and effort with fewer patients when they had to care less patients.

To the best of our knowledge, no study has investigated the relationship between the type of hospital room and incidence of HAP. We found that the risk of HAP was approximately three times higher in patients who stayed in rooms with more than four beds than that in those who stayed in rooms with three or fewer beds. According to a meta-analysis, using a single-patient room reduced healthcare-associated colonization of multidrug-resistant pathogens by 0.52 times and bacteremia rate by 0.64 times compared with using a multiple-patient room [32]. Patients who stay in single-patient rooms would have a lower risk of HAP as they have reduced direct or indirect contact with the reservoir compared with those who stay in multiple-patient rooms.

As a caregiver who is not a specialist revealed problems in the quality of care, infection, and safety, the need for fundamental alternatives for private nursing has been raised [33, 34]. Then, it was believed that the provision of specialized nurses contributed to reducing the incidence of HAP by minimizing various infection issues caused by the immature and inconsistent quality of care from nonprofessional caregivers [34]. However, no study has evaluated this issue. In South Korea, wards without caregivers were implemented in 2013. We found that patients who stayed in a ward with a caregiver had a 1.19-fold higher risk of HAP than those who were cared for only by nurses. It might be important to educate caregivers and patients regarding hand hygiene and other preventive behaviors to reduce the risk of HAP. Moreover, providing care by nurses without caregivers to patients who have a relatively higher risk of HAP would be necessary.

This study had some limitations. First, HAP defined by claim codes has limited accuracy and validity. In this study, we tried to use an operational definition of HAP that fitted the definition of existing guidelines, but we cannot exclude misclassification of HAP. Furthermore, diagnoses based on claims can differ from clinical diagnose. The HIRA database, however, is routinely audited, and the data are considered reliable and have been used in numerous peer-reviewed publications [35, 36]. Second, as the HIRA database includes claims for the entire hospital admission period, it was not able to establish the temporal relationship among the factors. For example, while MV is a well-known risk factor for HAP, patients might have MV due to HAP rather than vice-versa. Further longitudinal observational studies are necessary to confirm this finding. Third, our results may not be generalizable to other countries with different healthcare systems. Lastly, the patient samples in the HIRA dataset included linked data to claims accumulated over a year-long cycle, but patient data could not be linked across years. Therefore, it is not possible to conduct research that requires long-term follow-up of patients with our data. In the case of repeated hospitalizations within the same year, we included only the first hospitalization in the analysis. This approach may underrepresent the hospitalizations of high-risk patients, such as elderly patients or patients with multiple comorbidities, who are more likely to have multiple hospitalizations.

Despite these limitations, this nationwide study revealed the HAP incidence rate and identified factors associated with the incidence of HAP. This study confirmed the evidence on factors well-known in existing studies [8, 37] and additionally found that sociodemographic and hospital environmental factors might be related to the incidence of HAP.

## Conclusions

The incidence of HAP was associated with various sociodemographic, clinical, and hospital environmental factors. Taking a comprehensive approach to prevent and manage HAP is important. Thus, health professionals should work with various stakeholders, such as hospital management personnel and policymakers, to develop strategies to reduce HAP.

## Supplementary Information

**Additional file 1: Table S1.** ICD, KNHI procedure, and HIRA codes for all variables.

**Additional file 2: Table S2.** Comorbidity factors associated with the onset of hospital-acquired pneumonia.

## Data Availability

All the data used in this study are publicly available and properly cited. However, more guided instruction to get access to the data for transparency and reproducibility will be provided on request.

## Abbreviations

CI: Confidence interval
CKD: Chronic kidney disease
COPD: Chronic obstructive lung disease
HAP: Hospital-acquired pneumonia
HIRA-NIS: Health insurance review and assessment service-national inpatient sample
ICU: Intensive care unit
IQR: Interquartile range
MV: Mechanical ventilation
SD: Standard deviation
OR: Odds ratio
VAP: Ventilator-associated pneumonia
