# Supplementary material for: Comprehensive risk assessment for hospital-acquired pneumonia: sociodemographic, clinical, and hospital environmental factors associated with the incidence of hospital-acquired pneumonia
Source: BMC Pulm Med. 2022 Jan 12;22:21. doi: 10.1186/s12890-021-01816-9 (PMC8753882; doi:10.1186/s12890-021-01816-9)
Supplement: Supplementary file 1 — Additional file 1: Table S1. ICD, KNHI procedure, and HIRA codes for all variables. [file 12890_2021_1816_MOESM1_ESM.docx]

**Additional file 1**

Comprehensive risk assessment for hospital-acquired pneumonia: sociodemographic, clinical, and hospital environmental factors associated with the incidence of hospital-acquired pneumonia

Bo-Guen Kim, Minwoong Kang, Jihyun Lim, Jin Lee, Danbee Kang, Minjung Kim, Jinhee Kim, Hyejeong Park, Kyung Hoon Min, Juhee Cho, Kyeongman Jeon

**Table S1.** ICD, KNHI procedure, and HIRA codes for all variables

| **Variables** | **Codes** |
| --- | --- |
| **Pneumonia** | ICD-10 codes J12–J18, J85.1, and J85.2 |
| **Community acquired pneumonia** | ICD-10 codes J40, J209, J219, R05, R060, and R509 |
| **Underwent chest radiography** | ICD-10 codes G2101–G2105 |
| **Asthma** | ICD-10 codes J45 and J46 |
| **COPD** | ICD-10 codes J43 and J44, except for J430 |
| **Other chronic lower respiratory disease** | ICD-10 codes J40–J43 and J47 |
| **CKD** | ICD-10 codes N18 and N19 or KNHI procedure codes for dialysis or kidney transplantation: O7020, O7075, O9991, and R3280 |
| **Anemia** | ICD-10 codes D50, D539, and D649 or iron preparations and antianemic agents |
| **Tube feeding** | KNHI procedure codes Y7000, Y7001, Q2660, and Q2661 |
| **Suctioning** | KNHI procedure code M0137 |
| **Positioning** | KNHI procedure code M0143 |
| **Surgery** | KNHI procedure codes 05001 and 05011 |
| **Mechanical ventilation (for more than 3 hours)** | KNHI procedure codes M5857, M5858, and M5860 |
| **ICU admission** | HIRA codes AJ001–AJ590900 |
| **Bed-to-nurse ratio (nursing staff level)** | HIRA codes AB10–AB44 |
| **Types of hospital rooms (number of beds per room)** | HIRA codes AB10–AB44 and AO10–AO34 |
| **Ward without caregiver** | HIRA codes AO100–AO340 |

ICD, International Classification of Diseases; KNHI, Korea National Health Insurance; HIRA, Health Insurance Review & Assessment Service.
