# Supplementary material for: Comprehensive risk assessment for hospital-acquired pneumonia: sociodemographic, clinical, and hospital environmental factors associated with the incidence of hospital-acquired pneumonia
Source: BMC Pulm Med. 2022 Jan 12;22:21. doi: 10.1186/s12890-021-01816-9 (PMC8753882; doi:10.1186/s12890-021-01816-9)
Supplement: Supplementary file 2 — Additional file 2: Table S2. Comorbidity factors associated with the onset of hospital-acquired pneumonia. [file 12890_2021_1816_MOESM2_ESM.docx]

**Additional file 2**

Comprehensive risk assessment for hospital-acquired pneumonia: sociodemographic, clinical, and hospital environmental factors associated with the incidence of hospital-acquired pneumonia

Bo-Guen Kim, Minwoong Kang, Jihyun Lim, Jin Lee, Danbee Kang, Minjung Kim, Jinhee Kim, Hyejeong Park, Kyung Hoon Min, Juhee Cho, Kyeongman Jeon

**Table S2.** Comorbidity factors associated with the onset of hospital-acquired pneumonia

| **Variables** | **Group** | | **P-value** | **Multivariable** |
| --- | --- | --- | --- | --- |
|  | **No HAP** | **HAP** |  | **Adjusted OR (95 % CI)** |
|  | **(n = 486,909)** | **(n = 25,369)** |  |  |
| Myocardial Infarction | 6897 (1.4) | 533 (2.1) | <0.001 | 0.86 (0.78 - 0.94) |
| Congestive Heart Failure | 29751 (6.1) | 2689 (10.6) | <0.001 | 0.91 (0.87 - 0.95) |
| Peripheral Vascular Disease | 39386 (8.1) | 2417 (9.5) | <0.001 | 0.79 (0.75 - 0.83) |
| Cerebrovascular Disease | 49368 (10.1) | 4249 (16.8) | <0.001 | 0.91 (0.88 - 0.95) |
| Dementia | 28931 (5.9) | 4403 (17.4) | <0.001 | 1.32 (1.27 - 1.38) |
| Connective Tissue Disease | 14828 (3.1) | 772 (3.0) | 0.984 | 0.99 (0.91 - 1.07) |
| Peptic Ulcer Disease | 70973 (14.6) | 3727 (14.7) | 0.613 | 0.82 (0.79 - 0.86) |
| Mild Liver Disease | 84945 (17.5) | 3769 (14.9) | <0.001 | 0.76 (0.73 - 0.79) |
| Diabetes without complications | 100291 (20.6) | 6065 (23.9) | <0.001 | 0.82 (0.80 - 0.85) |
| Diabetes with complications | 40292 (8.3) | 2574 (10.2) | <0.001 | 0.84 (0.80 - 0.88) |
| Paraplegia and Hemiplegia | 6466 (1.3) | 820 (3.2) | <0.001 | 1.15 (1.05 - 1.25) |
| Moderate or Severe Liver Disease | 3000 (0.6) | 121 (0.5) | 0.006 | 0.66 (0.55 - 0.80) |
| Metastatic Carcinoma | 11608 (2.4) | 681 (2.7) | 0.002 | 1.15 (1.06 - 1.25) |
| Cancer | 74674 (15.3) | 3095 (12.2) | <0.001 | 0.90 (0.86 - 0.94) |

The multivariable analysis included age, sex, poverty, asthma, COPD, other chronic lower respiratory diseases, CKD, anemia, tube feeding, suctioning, positioning, surgery, mechanical ventilation, ICU admission, year of hospitalization, location of the hospital, and type of hospital.

HAP, hospital-acquired pneumonia; OR, odds ratio; CI, confidence interval.
